# Interactions Between Perioperative Anesthesia Management and Gut Microbiota: Current Research Advances

**DOI:** 10.1007/s10571-026-01701-1

**Published:** 2026-02-18

**Authors:** Naiqi Jiang, Lei Wang, Xiaodi Chen, Cuicui Yu

**Affiliations:** 1 School of Anesthesiology, Shandong Second Medical University, Weifang, 261053 China; 2 Department of Neurology, The Affiliated Yantai Yuhuangding Hospital of Qingdao University, No.20 Yuhuangding East Road, Yantai, 264000 China; 3 Department of Anesthesiology, The Affiliated Yantai Yuhuangding Hospital of Qingdao University, No.20 Yuhuangding East Road, Yantai, 264000 China

**Keywords:** Dysbiosis, Gut-brain axis, Surgical stress, Postoperative complications

## Abstract

The gut microbiota (GM), a complex and diverse microbial community residing in the digestive tract, plays a pivotal role in human health and disease. Recent studies have revealed a strong association between the GM and overall health, with dysbiosis potentially contributing to conditions such as inflammation, infection, and obesity. As medical technology advances, anesthesia has become indispensable in surgical and clinical procedures. Emerging evidence highlights the bidirectional interactions between the GM and anesthesia, which may exert profound effects on human health. This review synthesizes the current knowledge on the mutual influences of perioperative anesthesia and the GM, discusses the potential mechanisms underlying anesthesia-induced dysbiosis, and proposes strategies for preventing and managing microbiota imbalances. Future research should prioritize elucidating the precise mechanisms of anesthetic modulation of the GM and exploring microbiota-targeted interventions with the aim of potentially optimizing postoperative recovery and long-term health.

## Introduction

Trillions of microbial organisms coexist in the gut microbiota (GM), which is a dynamic community essential for host health (Mohajeri et al. 2018). Gut dysbiosis (GD) or microbial imbalance may adversely impact host health (Kawabata et al. 2022; Moya-Alvarez and Sansonetti 2022). The microbiota-gut-brain axis (MGBA) conceptualizes the bidirectional communication between these microbes and the central nervous system, influencing neurobehavioral and physiological outcomes (Cryan and Dinan 2012). Recent advances have deepened our understanding of the interplay between GM and health, revealing that gut microbes may influence brain function and behavior and contribute to diseases such as inflammation, infections and obesity through immune, neural and, metabolic pathways (Levy et al. 2017; Cryan et al. 2019; Fan and Pedersen 2021).

Simultaneously, the paradigm of perioperative care is evolving toward greater personalization, aiming to optimize outcomes such as pain management, recovery quality, and patient satisfaction. Within this context, a compelling body of evidence points to a significant bidirectional relationship between the GM and the effects of anesthesia. Nearly all surgical procedures involve anesthetic agents, however, the influence of the patient’s microbial community on anesthetic efficacy and perioperative pathophysiology remains an emerging frontier.

This review synthesizes recent advances in understanding the interactions between the GM and anesthesia, evaluates their implications for human health, and explores their clinical significance.

## Impact of Perioperative Anesthetic Factors on Gut Dysbiosis

### Common Pathophysiological Pathways

Multiple perioperative factors converge to disrupt GM homeostasis via shared pathways, primarily involving: (1) activation of the hypothalamic-pituitary-adrenal (HPA) axis and sympathetic nervous system, leading to systemic stress responses; (2) induction of local and systemic inflammation via immune cell activation and inflammatory mediator release; and (3) modulation of the MGBA, altering intestinal barrier (IB) function, microbial metabolite profiles, and neuro-immune crosstalk. These interconnected mechanisms form the basis for the dysbiosis observed across diverse perioperative insults.

### Preoperative Effects

#### Preoperative Anxiety

Preoperative anxiety is a common emotional response in patients who are awaiting surgery. Patients undergoing general anesthesia or those with preexisting anxiety disorders are particularly susceptible to heightened preoperative anxiety (Katsohiraki et al. 2020). Clinical studies have indicated that GM alterations occur in emergency patients as early as the day of admission, characterized by an increase in pathogenic bacteria. Even individuals exposed to traumatic events without physical injuries exhibit reduced levels of obligate anaerobes and Lactobacillus (Hayakawa et al. 2011). Xue Zhou’s team demonstrated in a rodent model that early exposure to anesthesia/surgery correlates with long-term GD and anxiety-like behaviors, mediated via MGBA (Zhou et al. 2023). Preoperative anxiety may dysregulate the GM, promoting pathogenic bacterial overgrowth (Deakin 1998), reducing beneficial species, and triggering a vicious cycle that exacerbates both dysbiosis and anxiety.

#### Preoperative Fasting

Preoperative fasting is a routine practice to reduce the risk of aspiration. Experimental evidence from animal models suggests potential additional benefits of this approach. Huang Wenfang et al. demonstrated in a murine intestinal ischemia/reperfusion (I/R) model that preoperative fasting preserves microbial homeostasis, increases levels of the microbial metabolite petroselinic acid, enhances IB function and improves survival rates (Huang et al. 2022). This is supported by direct experimental evidence showing that sevoflurane alters the GM and impairs cognitive performance in rodent models (Wen et al. 2020; Han et al. 2024). Thus, in animal models of intestinal I/R, preoperative fasting has shown benefits in preserving microbial homeostasis. However, the clinical relevance of these findings remains uncertain, as prolonged preoperative fasting is common and may theoretically disrupt microbial balance and promote inflammation.

#### Sleep Deprivation

Preoperative sleep insufficiency is common among elderly patients (Leung et al. 2015). Inadequate sleep before surgery is associated with poor postoperative recovery, increased complications, and prolonged hospital stays (Sibley et al. 2025). A study investigating sleep deprivation in preschool children found an increased abundance of Bacteroides and Bacteroidetes, as well as alterations in abundance ratios (Wang et al. 2022a). Animal studies have revealed that preoperative sleep deprivation induces postoperative GD and aggravates immunosuppression (Wang et al. 2022b). These findings indicate that sleep deprivation disrupts the microbial balance and impairs immune function.

#### Preoperative Anemia

Iron deficiency is the primary cause of preoperative anemia (Clevenger and Richards 2015). Iron deficiency anemia induces GD in mice, characterized by reduced microbiota diversity, decreased abundance of Muribaculaceae, and increased enrichment of Bacteroides (Sun et al. 2023). Anemia may reduce gastrointestinal mucosal perfusion, predisposing patients to dysbiosis (Malesza et al. 2022). Preoperative correction of anemia improves intraoperative hemodynamic stability (Musallam et al. 2011).

### Intraoperative Effects

#### Anesthetic Agents

Emerging preclinical data suggest that anesthetic agents differentially affect the GM. For instance, Ci Han’s team observed that sevoflurane inhalation anesthesia was associated with significant alterations in the GM composition in mice, reducing microbial diversity post-anesthesia (Han et al. 2021). In contrast, intravenous agents such as propofol and ketamine have shown minimal effects on the GM in animal models (Guo et al. 2021; Zhang et al. 2023). Animal studies have further indicated that dexmedetomidine may mitigate intestinal inflammation, reduce epithelial permeability, and help preserve IB integrity, thereby potentially stabilizing microbial communities rather than inducing dysbiosis (Feng et al. 2024). Consequently, these preclinical findings suggest a potential risk of postoperative dysbiosis associated with inhalational anesthetics, warranting further investigation. Future clinical studies are needed to determine whether monitoring and addressing such risks may improve patient outcomes.

#### Intraoperative Fluid Management

Preoperative fasting mandates intraoperative fluid resuscitation to maintain hemodynamic stability in patients undergoing surgery. Preclinical studies provide a mechanistic rationale for investigating fluid management in this context: animal models of intestinal I/R injury reveal that gut microbial diversity declines following I/R but improves with transfusion-mediated perfusion restoration (Kelly et al. 2021; Hu et al. 2022; Tong et al. 2024). Emerging, yet correlative, clinical data suggest a potential link. Studies comparing Goal-Directed Fluid Therapy (GDFT) and Non-Restrictive Fluid Therapy (NRFT) report associations where GDFT enriches microbial taxa linked to metabolic and coagulation recovery, whereas NRFT favors opportunistic pathogens (Pang et al. 2017; Bahlmann 2019; Fislage et al. 2023).

Therefore, the current evidence points to a promising yet unvalidated research direction. The optimization of fluid strategies, such as GDFT, to sustain intestinal perfusion and stabilize the microbiota represents a compelling future research priority. However, translating these findings into clinical practice requires direct validation through prospective studies designed specifically to establish causal relationships between fluid management, microbiota shifts, and hard postoperative outcomes in patients.

#### Anesthetic Depth and Surgical Approach

The Bispectral Index (BIS) is a processed electroencephalogram parameter used to monitor the depth of anesthesia (Singh 1999). Quan et al. reported that deep anesthesia (BIS target 30–45) in elderly patients undergoing abdominal surgery correlates with reduced short-term postoperative cognitive dysfunction and attenuated inflammatory cytokine release compared to light anesthesia (BIS target 45–60) (Quan et al. 2019). In addition to anesthetic agents, surgical techniques significantly modulate the GM. For example, abdominal surgeries for conditions such as irritable bowel syndrome (IBS) alter gastrointestinal motility, thereby reshaping microbial profiles (Moloney et al. 2016). In coronary artery bypass grafting requiring cardiopulmonary bypass (CPB), CPB-induced GD disrupts IB function and triggers systemic inflammation (Besser and Klein 2010; Pouard and Bojan 2013; Permanyer et al. 2019; Oyeyemi et al. 2022; Zhang et al. 2024b). Anesthesiologists must tailor anesthetic protocols to the type of surgery, with abdominal procedures exerting the most pronounced effects on microbial equilibrium. The interplay between surgical methods and anesthetic regimens differentially impacts GM homeostasis, necessitating personalized perioperative strategies.

## Effects of Microbiota Dysbiosis on Perioperative Anesthesia

### Impact of Gut Microbiota Dysbiosis on Postoperative Cognitive Dysfunction

Postoperative cognitive dysfunction (POCD) refers to the decline in cognitive function of the central nervous system (CNS) following anesthesia/surgery, and is a common complication affecting elderly patients postoperatively (Lian et al. 2021; Fislage et al. 2023). Perioperative factors, including surgery itself, antibiotics, opioids, and acid-suppressing medications, have been associated with alterations in the GM, which may lead to dysbiosis and consequent systemic and neuroinflammation implicated in cognitive impairment (Jiang et al. 2019). Anesthesia/surgery disrupts GM homeostasis, leading to altered bacterial composition, changes in fecal metabolites related to tryptophan and neurotransmitter metabolism (e.g., kynurenic acid, glutamic acid) and subsequent cognitive impairment (Lian et al. 2021). Studies have also suggested that preoperative anxiety may be a predictive factor for postoperative cognitive dysfunction (Oyeyemi et al. 2022) and could exacerbate GD, forming a vicious cycle: preoperative anxiety-GD-POCD. Therefore, within the conceptual framework of the MGBA, early identification and intervention for preoperative anxiety may be a potentially critical strategy for mitigating the risk of POCD.

### Impact of Dysbiosis on Postoperative Pain

The GM directly modulates pain sensitivity. GD activates immune cells and regulates neuroinflammation through signaling molecules (microbial metabolites, neurotransmitters, and neuromodulators), directly or indirectly regulating the excitability of primary nociceptive neurons and promoting peripheral and central sensitization to postoperative pain (Guo et al. 2019; Minerbi and Shen 2022). The severity of postoperative pain and the amount of analgesic medication used in patients undergoing upper limb surgery were significantly associated with the diversity of the GM and the abundance of specific bacterial genera (such as Collinsella and Lachnospira), suggesting that the GM could be a potential target for future research on postoperative pain (Brenner et al. 2021). Preoperative anxiety significantly influences postoperative pain intensity, particularly in gastrointestinal, obstetric, and gynecological surgeries (Álvarez-García and Yaban 2020; Zhang et al. 2021a). Preoperative anxiety-induced GD exacerbates microbial instability, aggravating postoperative pain, whereas pain further disrupts GM balance, forming another vicious cycle: preoperative anxiety-GD-postoperative pain.

### Impact of Dysbiosis on Immune Responses

A balanced GM is fundamental to immune maintaining homeostasis. Dysregulated GM compromises IB integrity, reduces beneficial metabolites (indole derivatives), and weakens immune function (Kinashi and Hase 2021). This immune dysregulation exacerbates systemic inflammation and increases susceptibility to postoperative infection. Additionally, altered GM may modulate host responses to anesthetics and analgesics, further complicating perioperative management of patients. Chronic dysbiosis is associated with metabolic and neurological disorders, which may elevate perioperative risks (Marietta et al. 2020; Zhang et al. 2021b; Zhu et al. 2024). These key alterations in gut microbiota composition associated with different surgery-related conditions are summarized in Table 1.

**Table 1:** Changes in gut microbiota associated to surgery-related conditions

Surgery-related condition	Microbiota changes	Subjects	References
POCD	↓ Relative abundance of *Lactobacillus*, irmicutes ↑ Relative abundance of *Bacteroides*, Proteobacteria	Mice	(Jiang et al. 2019; Han et al. 2021; Lian et al. 2021)
Postoperative Pain	↓ Relative abundance of *Collinsella* and *Coprobacter*	Human	(Brenner et al. 2021)
Preoperative Anxiety & Stress	↓ Relative abundance of Firmicutes ↑ Relative abundance of Proteobacteria	Rat	(Zhou et al. 2023)
	↓ Relative abundance of Firmicutes ↑ Relative abundance of Proteobacteria.	Human	(Jiang et al. 2015)

↑: indicates an increase; ↓: indicates a decrease. *POCD* postoperative cognitive dysfunction; *FMT* fecal microbiota transplantation

## Mechanisms of Anesthesia-Induced Gut Dysbiosis

Numerous medications, including oral drugs, can disrupt the GM and potentially lead to adverse health outcomes. Although this phenomenon has not been exclusively studied in inhalational anesthetics, it highlights the broader potential of pharmacological agents to alter gut microbial communities (Maier et al. 2018; Forslund et al. 2021). Perioperative anesthesia may induce dysbiosis, resulting in GM alterations in the host. These microbial shifts can modulate host behaviors, such as social activity, stress responses, and anxiety-related reactions (Chu et al. 2019). Therefore, understanding how anesthetics influence the GM is critical for improving postoperative recovery and long-term health.

### Direct Antimicrobial Effects

Inhalational anesthetics, such as isoflurane and sevoflurane, have been shown to induce significant GM changes in murine models (Jiang et al. 2019; Han et al. 2021). Intravenous anesthetics, such as etomidate, also exhibit antibacterial activity against gut bacteria (Cherfan et al. 2012; Raines 2015). In vitro experiments have demonstrated that sevoflurane exerts antimicrobial effects on both Gram-positive and Gram-negative bacteria, including multidrug-resistant strains (Han et al. 2021). However, studies have emphasized that these antimicrobial properties, whether direct or indirect, are not the primary mechanism of action for anesthetics, and their antibacterial efficacy remains minimal compared to their CNS effects.

### Impact of the Gut-Brain Axis

The GM, which resides in the gastrointestinal tract of mammals, extends the host’s digestive, metabolic, immune, and neurological functions (Cui et al. 2018). The concept of the MGBA has emerged to describe the complex and continuous signaling between gut microbes and the host nervous system (Bauer et al. 2016). Gut microbes play a pivotal role in the MGBA. Studies have integrated the CNS, autonomic nervous system, enteric nervous system, gastrointestinal tract, and diverse GM into a unified framework termed the MGBA. Research suggests that sevoflurane’s indirect effects on the GM may be mediated via this axis (Han et al. 2021). Bidirectional signaling between the MGBA involves bidirectional afferent (ascending) and efferent (descending) pathways, engaging brain regions such as the somatosensory cortex, insula, amygdala, anterior cingulate cortex, and hippocampus. Dysregulation of the MGBA is implicated in the clinical manifestations of IBS, including pain, altered bowel motility, and psychological dysfunction (Coss-Adame and Rao 2014). Metabolites produced by GM regulate blood-brain barrier integrity and participate in gut-brain communication by modulating anti-inflammatory factors in the signaling pathways (Sampson and Mazmanian 2015; Bosi et al. 2020; Han et al. 2021; Deng et al. 2021). Preclinical evidence suggests that these microbes can influence the CNS, which in turn may shape gut physiology and microbial composition through top-down regulation via the HPA axis and inflammatory responses (Coss-Adame and Rao 2014). Growing research on inflammatory mediators and the immune system has identified pro-inflammatory molecules linked to CNS disorders, such as interleukin (IL)−6, tumor necrosis factor-α (TNF-α), and the NOD-like receptor protein 3 (NLRP3) inflammasome complex (Agirman et al. 2021). Inhalational anesthetics, such as sevoflurane, may upregulate NLRP3 inflammasome-associated proteins in the gut and brain, triggering neuroinflammation and synaptic damage (Cattaneo et al. 2017). Studies have demonstrated that host physiology and behavior are modulated by gut microbial metabolites that enter the entering systemic circulation. This bidirectional signaling involves bottom-up pathways (afferent fibers to the CNS) and top-down pathways (efferent fibers to intestinal smooth muscle cells), analogous to feedback regulation (Bauer et al. 2016). Although anesthetics are rapidly eliminated post-inhalation, they exert transient “switch-like” effects on the GM, with impacts persisting until homeostasis is restored (Han et al. 2021).

**Fig. 1:**
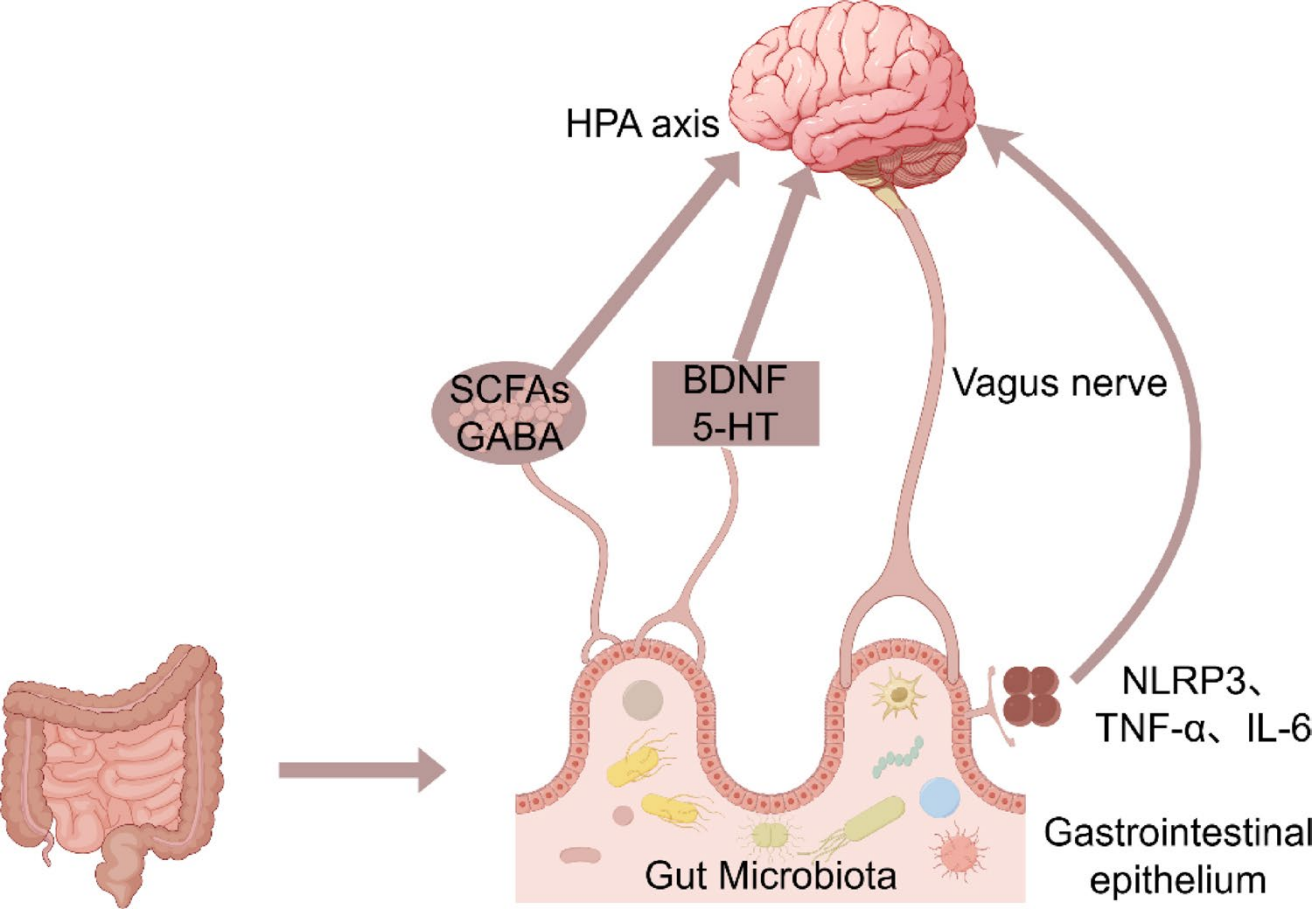
Gut-Brain axis: Microbial-Neuro-Immune interaction network. *5-HT *5-Hydroxytryptamine; *BDNF *Brain-Derived neurotrophic factor; *GABA* Gamma-Aminobutyric acid; *HPA axis* Hypothalamic-Pituitary-Adrenal axis; *IL-6* Interleukin-6; *NLRP3* NOD-like receptor family pyrin domain containing 3; *SCFAs* Short-Chain fatty acids; *TNF-α* tumor necrosis factor-alpha

The GM regulates neural pathways and the immune system via metabolites, whereas the gastrointestinal epithelial barrier maintains the microbiota-host balance. Together, these interactions form a dynamic microbial-neuro-immune network central to the gut-brain axis (Fig. 1). The original figure was created using figdraw.com and has been authorized for use.

### Immunomodulatory Mechanisms

The long-term effects of isoflurane on the GM may be correlated with neurodevelopmental toxicity (Wang et al. 2019). The chemical landscape of the brain, which determines environmental perception and response, is critically influenced by gut microbial metabolites. Using murine models, Chu et al. demonstrated that GM depletion significantly alters gene expression in excitatory neurons, glial cells, and other cell types within the medial prefrontal cortex (Chu et al. 2019). Evidence shows that a beneficial partnership has evolved between symbiotic bacteria and the immune system. GM interacts with the immune system by secreting anti-inflammatory (IL-10) and pro-inflammatory (IL-17) molecules, regulating the balance between Th17 cells (pro-inflammatory) and regulatory T cells (Tregs) (immune-suppressive), which are pivotal in gut immunity (Round and Mazmanian 2009). The distinct effects of various inhalational and intravenous anesthetic agents on gut microbial composition, as observed in preclinical models, are detailed in Table 2.

**Table 2:** Changes in gut microbiota associated with anesthetics

Surgery-related condition		Microbiota changes	Animal model	References
Inhalational Anesthetics	Sevoflurane	↑ Relative abundance of *Bacteroides*, Proteobacteria ↓ Relative abundance of *Lactobacillus* (effects lasting up to 2 weeks)	Mice	(Jiang et al. 2019; Han et al. 2021; Lian et al. 2021)
	Isoflurane	↓ α-diversity ↑ Relative abundance of Proteobacteria, Actinobacteria ↓ Relative abundance of Firmicutes, Clostridiales	Mice	(Serbanescu et al. 2019)
Intravenous Anesthetics	Propofol	↓ Relative abundance of *Prevotella*, *Alloprevotella* and *Lactobacillus*	Rat	(Guo et al. 2021)
	Esketamine	Altered the abundance of *Adlercreutzia equolifaciens* and *Akkermansia*.	Mice	(Zhang et al. 2023)
	Dexmedetomidine	↓ Relative abundance of *Coprobacillus*	Mice	(Feng et al. 2024)
	Morphine	↓ α-diversity ↑ Relative abundance of *Enterococcus faecalis* ↓ Relative abundance of Bacteroidales	Mice	(Wang et al. 2018)

All data were derived from animal models. ↑: indicates an increase; ↓: indicates a decrease

## Prevention and Management of Anesthesia-Induced Gut Microbiota Dysbiosis

As highlighted above, there is a growing recognition of the importance of GM stability, with the MGBA being increasingly integrated into clinical practice. POCD in patients has been linked to GD, suggesting that regulating GM stability may serve as a novel therapeutic target for preventing POCD (Lian et al. 2021). GD may lead to various health issues, including digestive disorders, impaired immune function, and even mental health disturbances (Lynch and Pedersen 2016; Fan and Pedersen 2021). The targeted therapies for GD are as follows (Shanahan 2010).

### Dietary Adjustments and Probiotic Supplementation

Dietary modifications may alter the composition and function of the GM, suggesting the potential for both prevention and treatment of cardiovascular diseases (Bhatnagar 2015). Reducing the intake of high-sugar, high-fat, and processed foods may help inhibit the overgrowth of harmful bacteria.

Probiotic supplementation through fermented foods or supplements has been shown to increase the abundance of beneficial gut bacteria (Bordalo Tonucci et al. 2017). Jiang et al. demonstrated in an animal model that cognitive impairment associated with anesthesia/surgery-induced GM alterations could be mitigated by probiotics (Jiang et al. 2019). In animal models, probiotics have been observed to alleviate dysbiosis caused by anesthesia/surgery and mitigate subsequent cognitive impairment (Jiang et al. 2019). These promising preclinical findings suggest a hypothesis: probiotic supplementation might mitigate anesthesia-induced dysbiosis and potentially reduce the risk of POCD in elderly patients. However, this premise awaits validation in future clinical trials. Supporting this, the preventive administration of beneficial substances, such as Lactobacillus, has shown protective effects against neuroinflammation in aged mice (Jiang et al. 2019; Pan et al. 2023). Thus, probiotics may counteract the anesthesia-induced dysbiosis and improve cognitive outcomes.

Another crucial strategy involves the consumption of prebiotic-rich foods or increasing dietary fiber intake to provide nourishment for beneficial bacteria, promoting their growth (Walters 2014). This approach could indirectly support a healthy GM.

### Fecal Microbiota Transplantation

Fecal Microbiota Transplantation (FMT) involves transplanting GM from healthy donors to restore the microbial balance in patients, enhance IB function, and alleviate dysbiosis. Preclinical and preliminary clinical studies suggest that FMT may exert antidepressant effects by modulating the gut microbiota, including increasing its diversity. As noted in a review, observations of concurrent increases in microbial diversity and behavioral improvements have been reported in both depression model animals and some patients receiving FMT (Zhang et al. 2024a).

### Other Strategies

Preoperative factors, such as anxiety, fasting, and sleep disturbances, have been associated with GM disruption. Addressing these factors, for example, through interventions to alleviate preoperative stress, may help maintain microbial equilibrium, benefiting certain patients. Adopting healthy lifestyle habits, including regular sleep patterns, moderate exercise and stress reduction, has been correlated with better gut health in some studies (Michael T. Bailey et al. 2011; Wang et al. 2022b; Zhu et al. 2024). For patients with chronic intestinal inflammation or iron malabsorption, oral iron supplementation might exacerbate oxidative stress and mucosal damage; thus, intravenous iron therapy is often recommended (Stein et al. 2016; Aykut et al. 2019; Cheng et al. 2021; Huang et al. 2022; Zhu et al. 2023).

These interventions should be tailored to individual differences and conducted under medical supervision to ensure safety and efficacy. Severe dysbiosis may require advanced clinical intervention. Future research should focus on elucidating how specific inhalational anesthetics impact the GM and developing targeted strategies (probiotics and prebiotics) to mitigate these effects. An overview of these potential intervention strategies, their reported effects on the gut microbiota, and corresponding host outcomes is provided in Table 3.

**Table 3:** Strategies against anesthesia-induced gut microbiota dysbiosis

Intervention strategy	Effects on gut microbiota	Host outcomes	Subjects	References
Probiotics	Reversed anesthesia/surgery-induced dysbiosis; Restored abundance of key taxa (e.g., Lachnospiraceae, Ruminococcaceae, *Bacteroides*).	Prevented reference memory deficits in aged mice.	Mice	(Jiang et al. 2019)
	Induced significant genus-level shifts but no significant phylum-level changes in postoperative fecal microbiota.	Reduced incidence of POCD in elderly patients.	Human	(Wang et al. 2021)
Prebiotics	↑ Relative abundance of *Bifidobacterium*	Improved POCD; Attenuated neuroinflammation.	Rat	(Yang et al. 2018)
Synbiotics	Postoperative counts of *Bifidobacterium*, *Lactobacillus*, Enterobacteriaceae, *Bacteroides*, and *Clostridium* remained stable (no significant decline).	Reduced infectious complications; Improved bowel motility; Reduced postoperative abdominal distension.	Human	(Theodoropoulos et al. 2016)
FMT	Reversed morphine-induced dysbiosis (e.g., restored Firmicutes/Bacteroidetes ratio, reduced pathogenic bacteria, increased beneficial taxa).	Reversed morphine-induced IB damage and systemic inflammation.	Mice	(Banerjee et al. 2016)
	Preoperative low abundance of Lachnospiraceae/Ruminococcaceae and high *Bacteroides* were associated with CPSP.	FMT from pain-free donors reduced susceptibility to CPSP.	Mice & Human	(Yao et al. 2022)
Dietary & Lifestyle Modifications	Exercise: ↑ α-diversity, altered β-diversity, ↑ Firmicutes/Bacteroidetes ratio.	Exercise: Prevented exacerbated neuroinflammation and cognitive decline in metabolic syndrome rats.	Rat	(Feng et al. 2017)
	Fasting: Maintained α-diversity, prevented the *increase* in the Firmicutes/Bacteroidetes ratio, increased beneficial bacteria (e.g., *Akkermansia*), reduced potential pathogens.	Fasting: Attenuated intestinal ischemia/reperfusion injury.	Mice	(Huang et al. 2022)
	Stress/Sleep Management: Increased microbiota stability, reduced pro-inflammatory taxa.	Management: May help maintain microbial homeostasis.	Human	(Kurdi et al. 2021)

↑: indicates an increase. FMT: Fecal Microbiota Transplantation; IB: intestinal barrier; CPSP: chronic post-surgical pain

## Limitations

As discussed, GD is reversible, and restoring the microbial balance may mitigate anesthesia-related adverse effects. However, the current body of evidence has several significant limitations that must be acknowledged to accurately interpret the findings and guide subsequent research.

First, the predominance of preclinical evidence is a major constraint. Most mechanistic insights discussed herein are derived from animal studies. Although these models are indispensable for establishing causality and exploring biological pathways, the direct extrapolation of these findings to human physiology and clinical outcomes remains speculative.

Second, there are fundamental methodological challenges inherent in GM research that critically impact the interpretability and comparability of the findings. Most studies rely on 16 S rRNA gene sequencing, which quantifies total microbial DNA but cannot distinguish between metabolically active, dormant, or dead bacteria. Discrepancies between the “total” (DNA-based) and “active” (RNA-based) microbial communities have been documented, indicating that observed taxonomic associations may not reflect functionally relevant microbiota-host interactions (Aef et al. 2018). These methodological confounders make it difficult to discern true biological signals from technical artifacts and hinder meta-analyses of studies.

Third, considerable heterogeneity exists in the available human studies, which are often small-scale, observational, and lack standardization in patient populations and perioperative care pathways.

Finally, there is a lack of standardized and validated perioperative GM endpoints. There is no consensus on the optimal timing for sample collection, the most relevant biospecimen, or which specific microbial metrics are most clinically meaningful in the perioperative context.

## Future Perspectives

Current research on the interplay between GM and anesthetic agents remains in its early stages, requiring advanced methodologies such as multi-omics analyses to identify interactions between gut microbes, metabolites, and anesthesia. Future studies should focus on elucidating how specific anesthetic drugs perturb the GM and develop targeted interventions, such as probiotics, prebiotics, or dietary modifications, to counteract these disruptions. This review highlights the bidirectional relationship between the GM and anesthesia, emphasizing its clinical significance. By modulating the GM, it may be possible to enhance anesthetic efficacy, optimize postoperative recovery, and reduce complications. These insights could pave the way for novel therapeutic strategies in the perioperative care. Moving forward, research should prioritize the identification of anesthesia-specific microbial and metabolic targets, establishment of protocols to restore microbiota equilibrium, and improvement of postoperative outcomes. Such efforts will not only deepen our understanding of the gut-brain-anesthesia axis but also offer actionable solutions to minimize anesthesia-related morbidity and enhance patient recovery.

## Data Availability

No datasets were generated or analysed during the current study.
